# Annotations

**Published:** 1906-12-29

**Authors:** 


### ANNOTATIONS.

### The Liernur Drainage System.

The Rural District Council of Stansted, Essex, has obtained from the Local Government Board permission to purchase the "Liernur" drainage works which have been experimentally established in the area controlled by the Council during the last four years. The system upon which these works are conducted is new to this country, where water drainage is the rule, but is extensively used on the Continent for low-lying districts where a good fall of water cannot easily be obtained. The main principle of the system is the drawing of the sewage *up* by suction to a storage tank. Thus the station where the sewage is treated can be not only distant from the place drained, but may absolutely be at a higher level. The method is in use at that fashionable French seaside resort, Trouville-sur-Mer, and is peculiarly applicable to seaside places. The ordinary way of discharging sewage into the sea, even though the pipes extend a considerable way out, does not incline the visitor to indulge in sea-bathing, whereas an undefiled sea and shore would assuredly be an attraction to any watering-place. But the method seems to be equally applicable to any district where the configuration of the ground makes the ordinary style of withdrawing sewage by gravitation impossible. According to Mr. J. A. Jones, sanitary engineer to the Government of Madras, the Liernur system has other advantages not dependent on situation. Among these he enumerates the following: The sewers can be laid at a uniform depth, just sufficient to protect them from frost. As no flushing is required—the sewage being drawn through the pipes to the storage tank by vacuum suction—the system is suited to places where a water supply is unobtainable or insufficient. No road gratings or sewer-ventilating columns are required in the streets, as the air pumps suck the whole range free from gases; and as the air pressure is uniform, particles of sewage are not liable to adhere to the sides of the sewers, as they often do when these are flushed with water. Moreover, as the success of the system depends on perfect suction, a leak must at once be noticed, simply because the suction would be observed to fail. This would prevent that percolation of the subsoil which often contaminates the supply of drinking-water in a neighbourhood under the present method. This in itself is no slight advantage. The Liernur system appears also to be comparatively inexpensive. Mr. Francis Liernur, in a paper which he read at a conference of municipal engineers, stated that the actual capital cost of the scheme, as carried out at Stansted, was less than £2 per thousand people, while the working expenses amounted to less than a shilling per head per annum, this expenditure including the cost of lifting the total sewage thirty-five feet up to the high-level sewer. The actual cost of cleaning the sewers and house connections—removing all sewage, noxious gases and the like occupied twenty minutes' time and cost 10d.—10d. for cleaning out two miles of pipes. This sounds moderate enough, and it is evident that the people of Stansted are satisfied with the success of the system, seeing that, after a four years' trial of it, they have resolved to purchase it.

### The First English Pharmacopœia.

The first Pharmacopœia published in England was the London Pharmacopœia. This work appeared in 1618 under the sanction of the Royal College of Physicians, and was published exactly a hundred years after that body had obtained its charter. The college obtained legal recognition for its list of drugs, which was a mere compilation of ancient authors like other works of the period, from His Majesty James I. (and VI. of Scotland). The instrument called upon "All apothecaries of this realm to follow the Pharmacopœia" which was then compiled. This Pharmacopœia contained, like the modern ones, no indication of the purpose for which the medicines were intended. This shows the wisdom and prudence of the old members of the college; no allusion to the therapeutic action of the remedies was published until the year 1649, when Culpeper published his "Physical Directory" in English. This contained a translation of the London Pharmacopœia, and notes on the uses of the remedies were added whenever it was possible; but, as Culpeper himself confessed, the "virtues" of the drugs "were not easily gotten," and as quacks were plentiful at the time he was afraid that if he told all about them "knaves might put them in practice to do mischief." The first London Pharmacopœia was a handsome folio volume embellished with a carefully executed frontispiece of a high order of merit. The legal instrument which accompanied it, printed on its front page, "Straightly required all and singular apothecaries that they, every one of them, do not compound or make any medicines or medicinal receipt or prescription, or distil any oil or waters, or other extractions, in the waies described by any other book or dispensatories whatsoever. But after the onely manner and form that hereby is or shall be directed . . . upon pain of our high displeasure." This volume marks the character of the medical knowledge of the time, its origin, and its purposes, and certainly could only at any time have been compiled by a committee of medical men. It represented the extent of the knowledge then existing. In that sense it completely differs from the British Pharmacopœia, which really only represents a small number of the drugs employed by a medical man, and which is used by pharmacists and drug vendors rather than by practitioners of medicine. Several editions followed without much change of form or matter, and at short intervals, showing the readiness with which the work commanded a sale. This work existed up to 1851. Its success brought many competitors into the field who published, like Culpeper, editions of their own, and placed them on the market.

